# Exploring attitudes towards use of technology to support stroke survivors living at home: A quantitative and qualitative content analysis study in Spain

**DOI:** 10.1177/20556683211019690

**Published:** 2021-08-20

**Authors:** Leire Ortiz-Fernandez, Charlotte Magnusson, Agustin Gutierrez Ruiz, Lorea Martinez Indart, Joana Sagastagoya Zabala, Juan Andres Alava Menica, Eunate Arana Arri

**Affiliations:** 1 Physical Medicine and Rehabilitation department, Cruces University Hospital- Osakidetza-Basque Health Service, Barakaldo, Spain; 2 Rehabilitation Engineering and Design, Lund University, Lund, Sweden; 3 Biocruces Bizkaia Health Research Institute, Barakaldo, Spain

**Keywords:** Telehealth, stroke rehabilitation, decision making system, self-management, self-care

## Abstract

**Purpose:**

The aim of this study was to better understand the attitudes towards the use of technology to support chronic stroke survivors in a home-based setting.

**Methods:**

A quantitative study was used on the data obtained from a face to face survey with the sample group, incorporating quantitative statistical analysis.

**Results:**

Participants reported positive attitudes towards using technology for their own independent health management. The purpose of the home-based technology was different: source of information, supporting self-management, pharmacological treatment reminders… and differed according to age, educational level and survivor disability. Installing devices and sharing information remains a challenge.

**Conclusions:**

100% of stroke survivors living in the community with any type of disability, reported that they would like to use technology as a tool to help improve their health status.

## Background

Stroke is the leading cause of long-term disability in western countries^1,2^ resulting in life altering changes for both the stroke survivor and their closest family, sometimes, resulting in profound difficulties and needs. Different locations of the stroke cause different results. A stroke on the right side of the brain can lead to paralysis on the left side of the body, vision problems (and spatial unawareness – “hemispatial neglect”), quick, inquisitive behavioural style (including denial/unawareness), memory loss, a left sided stroke, on the other hand, may lead to paralysis on the right side of the body, speech/language problems, slow, cautious behavioural style (aware of the problems), memory loss as well. Fatigue is also a common problem after all brain injuries. Remaining symptoms after the stroke might cause difficulties in handling things with two or one hand, difficulty or inability to walk, complications such as falls, fractures, dysphagia, balance and dexterity issues, problems for understanding, speaking, reading, calculating and visual recognition, difficulty in perceiving and/or processing visual or auditory information, difficulty to start, follow and/or remember instructions and sequences….^3^

Most strokes are due to ischemic assault which kills one third of the patients leaving another third with severe disabilities. Fortunately, as a result of the improvement of the acute medical treatment, there’s an increasing number of stroke survivors.^4^

Surviving a stroke and living with its effects involves a long and challenging process for patients and their families including rehabilitation process, changes in the physical, social, emotional aspects, and furthermore the majority of these patients must follow a tight control of cardiovascular risk factors and life-style changes to prevent the risk of suffering a recurrent stroke.^5^

Limited data exists on the long-term needs of community-dwelling stroke survivors but for the technologies, scarcity of data is the norm. Prior qualitative research (via interviews and focus groups) on end user’s requirements for e-rehabilitation are published in the literature however, they do not consider a holistic point of view of living after stroke neither they focus the attention on the needs of new technologies in rehabilitation (robots, video games, telemedicine…) which should be adapted to the end user’s requirements.^6,7^

Nevertheless, there is an opportunity to explore previously unreported factors, particularly those related to technology (e-Health/m-Health) such as smartphones applications for improving healthy lifestyles, enhancing adherence to pharmacological and non-pharmacological treatment, empowering the patient and caregivers with self-management tools, tracking wearables to promote outdoor exercise or to detect complications (falls, arrhythmia, patient getting lost…), home-based telehealth, detecting the risk of a recurrent stroke in real time among other ideas.

As health care providers concerned with quality of life of stroke survivors, our aim was to **better understand the attitudes of chronic, home-based, stroke survivors regarding technology** (e-Health/m-Health). As a secondary objective, differences between age, disability and educational level were evaluated.

## Material and methods

### Research design

For this study, questionnaires were used to capture the required data to investigate and gather patients’ experiences and interest in potential technology which could be beneficial for chronic stroke survivors living at home with a disability.

Ethics committee approval was obtained on behalf of the STARR project registered in clinicaltrial.gov (NCT03580642).

### Participants and context

Post stroke chronic phase adults (both male and female) with any type of impairment, living within the community and able to voluntary participate and understand the instructions were identified and invited to participate in the outpatient neurorehabilitation consultation of a Cruces University Tertiary Hospital, Spain, from September to December 2017.

The objective and the purpose of the study were explained and the patient was free to read and sign the Informed Consent Form before the collection of any data, they were allowed to voluntarily withdraw their consent at any moment. Caregivers were welcome to attend the interview and also sign the consent to participate. Individuals were excluded if any of the following applied: unable to cope with the interview or with severe language and/or cognitive impairments assessed by Mississippi Aphasia Screening Test (<45) and Montreal Cognitive Assessment (MoCA <26) respectively. Participants were enrolled by a convenience sampling method. Participants did not receive any honorarium.

Given that age, educational and disability level have been reported as confounders in the literature, they were analysed separately. The sample was divided and compared the differences between age groups (group 1 = ≤45 years old, group 2 = 45–65 years old, group 3=≥65 years old), educational (primary, secondary and university education) and disability level measured by Barthel Index.

The researchers, 2 trained physical medicine rehabilitation (PMR) MDs, male and female, with a wide background in neurorehabilitation. The researchers were impartial and only had professional familiarity with the patients. The reasons and interests in the research topic were explained to the participants.

### Questionnaire administration and data collection procedure

The interview guide and the interview questions, were designed by researchers at Lund University, Sweden, CEA, France and Osakidetza, Spain, in collaboration with patient and care organization partners in the STARR project. The themes explored by the interviews were consequences of the stroke, daily activities before and after the stroke, adherence to treatment and position towards technology. The interview technique consisted of a questionnaire (online Appendix 1), including both close-ended and open-ended questions in one session. These questionnaires were translated and administered in Spanish which is the mother language of the participants.

The interview was held in a comfortable atmosphere in a consultation of outpatient setting of the Tertiary University Hospital with a face-to-face interview. The questionnaire was completed in 20–30 minute discussions moderated by the researcher. Participants were verbally asked pre-determined questions, to which they wrote down their responses, where necessary additional explanations were given. The results of the questionnaires were not returned to participants for further comment.

Data was extracted from medical records based on demographics, stroke features, vascular risk factors, medical care and planned rehabilitation, including previous functional situation, education level and impairment details.

Identifying information was removed from the documents and written notes were taken when necessary.

### Data analysis and statistical analyses

Researchers independently coded the data, developing a formal coding framework and categories which were completed after a second peer debriefing session. During the analysis process, when differences were observed, the researchers carefully examined any potential source of bias ensuring consistency. Researchers duplicated and verified the data in other following peer sessions.

Descriptive statistics were used to present socio-demographic data and medical information. Barthel index was used to categorize stroke severity as independent, mild, moderate and severe. Quantitative statistical analysis was utilised.

The qualitative variables were described in percentages and quantitative variables with median and range.

We analyzed the data with SPSS (version 23.0) statistical software package, testing subgroup differences using chi-square analysis or Fisher exact test. Reported P values are two-sided. Significance level was specified at 0.05.

## Results

### Participants characteristics

A total of 56 participants, including 22 with mild impairment in communication (14) and/or in cognition (8) fulfilled the inclusion criteria for this study. The majority of the participants were male with a mean age of 67.50 years. No refusal to participate nor drop-outs were reported. The main socio demographic factor characteristics and medical information are shown in Table 1.

**Table 1.** Demographical data and medical records.

Variable	No. (%)
No. of women/men	22 (39.28%)/34 (60.72%)
Mean age women/men	65.27 (35–88)/68.94 (38–88)
Group 1	5 (8.93%)
Group 2	16 (28.57%)
Group 3	35 (62.50%)
Ethnicity	54 Caucasian (96.42%) (Spain)
	2 Hispanic (3.58%) (Peru, Colombia)
Educational level	
– Primary school	27 (48.22%)
– High school	22 (39.28%)
– University	7 (12.5%)
Risk factors	
– Hypertension	36 (64.28%)
– Diabetes mellitus	13 (23.21%)
– Dyslipidaemia	26 (46.42%)
– Current smoker	9 (16.07%)
– Atrial fibrillation	19 (33.92%)
Stroke features	
– Ischemic	
○ Cardioembolic	14 (25%)
○ Atherothrombotic	19 (33.93%)
○ Others	23 (41.07%)
– Affected circulation	
○ Anterior	44 (78.58%)
○ Posterior	11 (19.64%)
○ Both	1 (1.78%)
– Affected side	
○ Right	18 (32.14%)
○ Left	32 (57.14%)
○ Both	6 (10.71%)
– Type of acute treatment	
○ Fibrinolysis	4 (7.14%)
○ Thrombectomy	10 (17.85%)
○ None	42 (75%)
Basal NIHSS	9.29 (1–26)
Number of medications: mean	9.69
– <5	7 (12.5%)
– 6–10	25 (44.64%)
– >11	24 (42.86%)

### Consequences of stroke

In terms of disability, all participants suffered a type of impairment, with a final Barthel index of 77.03.

Stroke has a large number of negative consequences on survivors’ everyday life. The participants in study talked about many physical difficulties, for example difficulties with mobility of their upper and/or lower limb as well as a general reduction of the physical activity, balance issues and fatigue.

93% were ambulatory to some extent, although some needed assistance such as a cane, crutch and/or foot-up orthoses.

Study participants also evoked communication problems, cognitive difficulties, anxiety, depression and emotionalism. They expressed a negative feeling about the new situation (Table 2).

**Table 2.** Consequences of stroke.

Employment situation previous to stroke (working/retired)	21 were of working age, (37.5%): 19 were employed. 35 were retired (62.5%)
Employment situation after stroke	From the 21 people who were in working age, none returned back to work
Basal Barthel index: mean	(25–100)
– Independent	54 (96.42%)
– Mild dependent	0
– Moderate dependent	1 (1.78%)
– Severe/totally dependent.	1 (1.78%)
Familiar situation before stroke	
– Living on their own	6 (10.71%)
– Living with relatives	50 (89.29%)
○ Needed help of a third person	1
Familiar situation after stroke	
– Living on their own	4 (7.14%)
– Living with relatives	52 (92.86%)
○ Needed help of a third person	16
Final Barthel index: mean	77.03 (10–100)
– Independent	14 (25%)
– Mild dependent	8 (14.28%)
– Moderate dependent	22 (39.28%)
– Severe/totally dependent.	12 (21.42%)
Consequences of stroke	
– Upper limb mobility difficulty	41 (73.21%)
– Walking difficulties	39 (69.64%)
– Balance problem	12 (21.42%)
– Communication problem	14 (25%)
– Swallowing problem	3 (5.35%)
– Perception problem (ie neglected limb) or attention problem	5 (8.92%)
– Memory loss	8 (14.28%)
– Emotional problem	6 (10.71%)
– Vision problem	4 (7.14%)
– Fatigue	17 (30.35%)
Able to walk independently	31 (55.35%)
Need assistance:	21 (37.5%)
– Wheelchair	4 (7.14%)
– Cane	14 (25%)
– Crutch	5 (8.92%)
– Foot up	6 (10.71%)
Their feeling about the new situation is negative	17 (30.35%)

### Rehabilitation

All the survivors needed rehabilitation (RHB) during their stay in the hospital with being 30% discharged to a RHB nursing home.

The most frequent combination of RHB were physiotherapy and occupational therapy. Mean average time of treatment was 9.8 months excluding the botulinum toxin A treatment periods (range 1 to 14 months) (Table 3).

**Table 3.** Rehabilitation features.

Care and support after hospital discharge	
– Discharged to a RHB hospital	17 (30.36%)
– Discharged home RHB	39 (69.64%)
RHB needs	
– PT	7 (12.5%)
– PT + OT	19 (33.93%)
– PT + OT + ST	18 (32.14%)
– OT	1 (1.78%)
– OT + ST	0
– ST	5 (8.92%)
– ST + PT	4 (7.14%)
– No supervised RHB	2 (3.57%)
– Average time excluding TBA injection patients	9.80 months
Complications:	
– Spasticity	11 (19.64%)
– Pain	17 (30.35%)
– Depression	6 (10.71%)
– Falls	13 (23.21%)

RHB: rehabilitation; PT: physiotherapy; OT: occupational therapy; ST: speech therapy; TBA: toxin botulinum A.

### Current situation

When describing life **before** stroke, participants often evoked working activities (90% of people in working age), domestic activities such as cooking, sport and hobbies, as well as social activities such as visiting family, friends or taking care of grandchildren.

When talking about life **after** stroke, patients mentioned activities such as dressing one’s self, grooming, eating, cleaning and preparing simple meals. These data show that they need a considerable time to do basic activities of daily life.

Walking was considered a very frequent and important activity after stroke as it gives autonomy and is part of the non-pharmacological treatment to keep the physical activity level and control cardiovascular risk factors (high blood pressure, dyslipidaemia, diabetes mellitus, and obesity). Social activities such as visiting friends and family or taking care of relatives was less frequent depicting life after stroke. On the contrary, they mentioned watching TV, reading activities that were not present in their discourses on life before stroke (Table 4).

**Table 4.** Current situation.

Daily activities	Before stroke	After stroke
Work	19 (33.92%)	0
Going for a walk	20 (35.71%)	29 (51.78%)
Basic daily activities^a^	2 (3.57%)	15 (26.78%)
Visiting family and friends	50 (89.28%)	10 (17.855)
Sports (therapeutic exercises)	20 (35.71%)	26 (46.42%)
Domestic activities	50 (89.28%)	10 (17.85%)
Watching TV	12 (21.42%)	24 (42.85%)
Reading	7 (12.5%)	9 (16.075)
Surfing the net	5 (8.92%)	8 (14.28%)
Traveling	3 (5.35%)	1 (1.78%)
Communication		
– Normal	56 (100%)	42 (75%)
– Affected	0	14 (25%)

^a^Basic daily activities include feeding, personal toileting, bathing, dressing and undressing, getting on and off a toilet, controlling bladder, controlling bowel, walking , climbing stairs or propelling a wheelchair if unable to walk.

### Technologies in stroke survivors

A vast majority of the participants (91.07%) were **familiar** with technologies especially with television (78.57%) and cell phone (71.42%). Tablet (17.85%) and tele-assistance (8.92%) were not very popular.

There was strong consensus (92.85%) about the **eagerness of using** technologies in a health care setting, however their perception of use was different. More than 70% of the participants would like to employ the technology for self-management and for gathering information (causes of stroke, how to identify a new stroke, what to do, different types of stroke, recovery time…).

**Figure 1.**
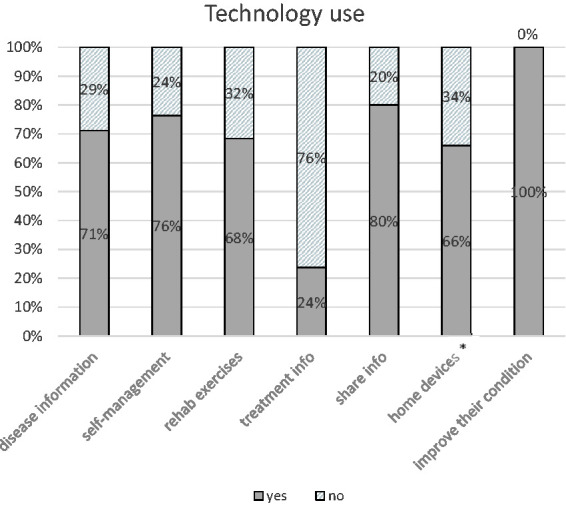
Technology use. *Home devices such as tensiometer, glucometer, pedalier, scale or even cameras and movement sensors.

The therapeutic **function** of the technologies related to health was divided in non-pharmacological (rehab exercises) and pharmacological treatment. More than half would be grateful to use it for rehab treatment exercises (i.e. exercises for upper limb such as prevention of painful shoulder, dexterity of the hand, coordination, safe balance exercises, aerobically exercise program, strength exercise program, stretching spastic muscles…) while only a quarter would like for treatment info such as medication reminders (i.e. “have you taken your medication?” “If response is negative, do not forget to take it before 8:00 pm”). All the survivors wanted to use the technology to improve their condition, increase their autonomy, self-efficacy and consequently their self-management (Figure 1).

Despite having a positive disposal for eHealth, the vast majority (89,5%) would accept it only for certain **activities** or issues.

Fortunately, a significant majority of stroke survivors affirmed following the medical recommendations and instructions for the pharmacological treatment only 5.35% did not.

With regard to **data sharing,** the majority of patients of the responders would allow sharing information with the relatives and/or health staff, as shown on Table 5.

**Table 5.** Data sharing.

	Yes	No	NA
Medical doctor	44 (78.57%)	11 (19.64%)	1 (1.78%)
Family	38 (67.85%)	11 (19.64%)	7 (12.5%)
Friends	18 (32.14%)	29 (51.78%)	9 (16.07%)
None	11 (19.64%)		
All	3 (5.35%)		

Whilst 19.64% would want to keep their data private.

Almost half of the participants would accept installation of devices at home (Table 6).

**Table 6.** Interview answers.

Already use technological devices	51 (91.07%)
Want to use technological devices in a health care setting	52 (92.85%)
Would like to use technology	
– For disease information	
○ yes	36 (64.29%)
○ no	15 (26.79%)
○ don’t know/depends	5 (8.92%)
– For auto-control	
○ yes	42 (75%)
○ no	13 (23.22%)
○ don’t know/depends	1 (1.78%)
Technology devices for treatment	
– For RHB + exercises	25 (44.64%)
– Pharmacologic treatment control	9 (16.07%)
– Everything	4 (7.14 %)
– Don’t know	18 (32.14%)
Ready to install gadgets at home	
– Yes	31 (55.36%)
– No	16 (28.57%)
– Don’t know	9 (16.07%)

#### Subgroup analysis by age

For technology as a disease **information source**, a dominant positive feeling emerged, as 100% of the younger patients, 86,7% of medium age and 59.4% of older age identified this function as useful. The interest in using technology to support **self-management** (100%; 87.50%; 67.60% respectively), for rehabilitation exercises (100%, 66.7% and 63.6% respectively) followed similar distribution. However, the distribution of percentages were different for **pharmacological treatment reminders** (0%, 25%, 27.3% respectively), but most of these differences are not statistically significant.

Similar point of views apply for the readiness to install devices at home 100% of the younger patients, 85% of medium age and 52% of older age (p = 0.025) being these differences statistically significant.

#### Subgroup analysis by educational level and disability level

There was a directly proportional relation between the educational level and the intention of **installing devices** at home being this tendency similar with the disposal for **sharing** information with third parties, in other words, those participants with higher level of education, such as university studies, showed greater tendency to install devices and to share information compared to those with no education (Table 7).

**Table 7.** Educational level.

	Info sharing	
	Yes	No
Primary school	86%	14%
Secondary school	95%	5%
University studies	100%	0%

There were no statistically significant differences with disability level and technology use, eagerness of using it, functionality and data sharing.

## Discussion

This is the first type of study in Spain to obtain detailed information on the **experience** and potential uses referring to new technologies (e-Health/m-Health) in the chronic stroke survivors in the home, focused on the experience of technologies can offer. Identifying the users’ attitude towards technology can help to define interventions to support best stroke survivors living in the community, reduce dissatisfaction, improve adherence to physical activity exercises, to medication intake and to improve quality of life of patients and caregivers indirectly. There are other domains for instance, everyday living, work, leisure, social support, driving and finances…. Although they can also be fulfilled with serious games, tele-rehabilitation, robotic devices, virtual reality, wearables/sensors, tablets, health devices and others.

Our results showed physical, cognitive and physiological problems, which might have a huge negative impact of their daily life. These findings are not new and correspond the reviewed literature.^3^

Previous studies on long-term unmet needs were concentrated on needs resulting from functional deficits after stroke, such as management of body function, participation in basic and instrumental activities of daily living, or secondary needs of new socio-familial or environmental factors.^8–11^ It is important to understand the specific needs perceived by stroke survivors for a patient-centered health and social care. Furthermore, the unsatisfied needs perceived by patients may differ from those perceived by healthcare professionals and caregivers.^7^ Furthermore, younger survivors potentially could have a higher functional needs to be fulfilled, not only home-rehabilitation exercises but also intellectual fulfilment, work, holiday and family support as they need a higher degree of recovery to participate in the society. Following this line of reasoning, clinicians may utmost focus on the emotional support and their skills to adapt to the new living situation rather than, clarifying repeatedly stroke’s functional recovery process. Definitely, rehabilitation efforts and reintegration on the society remains a challenge in this age group.^10,12^

In the study, a significant proportion of participants reported be **familiar** with technology, having a positive attitude towards its use in health-related basis. Notably, all of them wanted to improve their condition. This is not surprising as other studies have shown that patients, especially chronically ill patients, are positive towards being involved in their care and rehabilitation processes.^13^

The results of this study showed differences in the purpose of the technology. Attitude towards using technologies were most prominent in **home-rehabilitation exercise** program instead of cardiovascular risk factor control, maybe due to sampling recruitment in rehabilitation consultation basis. In addition, installing devices at home is convenient in younger patients (p < 0.05). Undeniable, patients are no longer passive^14,15^ receivers of care. The findings suggest higher involvement in the decision making process as they preferred to choose the aim of using the technology. For example, 76% would like to employ the technology for self-management, 71% for learning information about disease, medium age group in particular. However, when interviewed about treatment, they felt confident in pharmacological management, especially younger patients who showed dissatisfaction of pharmacological treatment reminders.

Nonetheless, it has been generally acknowledged for years that non-adherence rates for chronic illness regimens and for lifestyle changes are around 50%, admitting the success of a medical treatment is largely determined by compliance.^16,17^ Given that non-compliance of drug treatment may lead to complications is crucial to control the cardiovascular risk factors and to prevent a recurrent stroke. To foster adherence, patient centered approach is essential, listening to survivors and care-givers to discover the unmet needs and what is important for them.

For data sharing, results showed a notable variation in opinion which need to be evaluated in the context of an application, taking into consideration the aim of the information sharing.^18^

It is not completely clear whether and how technologies can be implemented in stroke rehabilitation in different settings but, it is worth considering flexibility in use of the technology so that it could allow personalization to varying abilities, interests and situations.^17^ These factors associated with unmet needs could help guide policy decisions, particularly for tailoring care and support services provided after discharge in home basis.^10^

STARR (The Decision SupporT and self-mAnagement system for stRoke survivoRs) project and the system developed in it are targeting the self-management of stroke risk factors. Existing predictive models of stroke risk will be used, a modular, affordable, and easy-to-use system to inform stroke survivors will be developed aiming to inform about the relation between their daily activities and the risk of having a secondary stroke leading to better prevention and reduction of secondary strokes and to a more efficient participation of survivors in medical decision-making process.

STARR project could be a solution to manage the attitude towards technology unsatisfied need considering age, disability level, educational level, home environment. The system should be flexible and able to engage patient’s participation increasing internal and external motivation.

### Limitations

Some limitations exist in the study, including modest sample size, aphasic population is not evaluated and needs to be considered.^18^ Those without rehabilitation consultation review such as the institutionalised or transient ischemic attack were not studied and may have different unmet needs. Subgroups are not homogeneous, it was not feasible to obtain young stroke survivors as the prevalence of this disease is not common within this age group. Time delay between data collection, analysis and publication exits, however, based on our experiences, and those gathered from colleagues and patients, the attitudes towards technology and responses to technology change at a slower than the technology itself and vary more on a generation basis.

## Supplemental Material

sj-pdf-1-jrt-10.1177_20556683211019690 - Supplemental material for Exploring attitudes towards use of technology to support stroke survivors living at home: A quantitative and qualitative content analysis study in Spain Click here for additional data file. Supplemental material, sj-pdf-1-jrt-10.1177_20556683211019690 for Exploring attitudes towards use of technology to support stroke survivors living at home: A quantitative and qualitative content analysis study in Spain by Leire Ortiz-Fernandez, Charlotte Magnusson, Agustin Gutierrez Ruiz, Lorea Martinez Indart, Joana Sagastagoya Zabala, Juan Andres Alava Menica and Eunate Arana Arri in Journal of Rehabilitation and Assistive Technologies Engineering
