# Supplementary material for: Exploring attitudes towards use of technology to support stroke survivors living at home: A quantitative and qualitative content analysis study in Spain
Source: J Rehabil Assist Technol Eng. 2021 Aug 20;8:20556683211019690. doi: 10.1177/20556683211019690 (PMC8381459; doi:10.1177/20556683211019690)
Supplement: sj-pdf-1-jrt-10.1177_20556683211019690 - Supplemental material for Exploring attitudes towards use of technology to support stroke survivors living at home: A quantitative and qualitative content analysis study in Spain [file sj-pdf-1-jrt-10.1177_20556683211019690.pdf]

Identifier :
Date :

STROKE SURVIVORS SEMI-STRUCTURED INTERVIEW GUIDE

PROFILE

1) Sex : ☐ F ☐ M Year of birth:

2) Education level :

☐ none ☐ primary ☐ secondary ☐ University

3) Home care by:

☐ Spouse ☐ Children ☐ Grandchildren ☐ Nephew / Niece

☐ Other: ……

4) When did the stroke happen?

………

5) What consequences of stroke do you feel in your daily life? How do you experience them?

………

………

DAILY ACTIVITIES CARRIED OUT BY THE STROKE PATIENT

6) Can you describe your typical day since your stroke?

………

………

………

………

………

………

………

This project has received funding from the European Union’s Horizon 2020 research and innovation programme under grant agreement No 689947

Identifier :
Date :

7) What are your main activities now? (Hobbies, reading, sports activities, games, etc.)

………

………

………

………

………

8) Do some of your activities take place outdoors?

………

………

9) What is the frequency of these activities? (Per day, per month, etc.)

………

………

10) Do you need assistance with these activities and if yes, what kind of assistance?

………

………

………

11) Do you have difficulties in the following activities :

- Daily activities (getting up, dressing, eating, etc.)

Before stroke:

………

………

After stroke:

………

………

- Communication (family, friends, etc.)

Before stroke:

………

………

After stroke:

………

………

This project has received funding from the European Union's Horizon 2020 research and innovation programme under grant agreement No 689947

Identifier :
Date :

- Other activities (hobbies, games, reading, etc.)

Before stroke:
……

……

After stroke:
……

……

ADHERENCE TO TREATEMENT

12) Do you forget to take your medication?
☐ Yes ☐ No

13) If yes, why (e.g. inattention, health improvement or degradation)?

……

……

14) Do you respect your physician's instructions for taking your medication and exercising (timetable, etc.)?
☐ Yes ☐ No

TECHNOLOGIES

15) Do you use "new technologies" (phone, computer, TV, etc.)? Which ones?

……

……

……

16) Would you like to use technologies providing you with useful information for your treatment? (Medication, exercises, activities, etc.)

……

……

……

17) Would you be interested in using technologies for keeping track of and helping you self-manage some of your activities?
☐ Yes ☐ No

18) If yes, which activities / tasks would you like to use these technologies for? (Taking medication, therapeutic exercise, etc.)

This project has received funding from the European Union's Horizon 2020 research and innovation programme under grant agreement No 689947

Identifier :
Date :

………

………

19) Would you be ready to install new technologies at home (or to wear them) in order to keep track of and self-manage your daily activities?

………

………

20) What other technologies could improve your daily life?

………

………

21) Would you be ready to share information?

☐ Yes ☐ No

If yes, with who? Family, medical doctor, health care staff, friends, nobody…

This project has received funding from the European Union's Horizon 2020 research and innovation programme under grant agreement No 689947
